# Serum Antithyroglobulin Antibody Levels Are Associated with Diabetic Retinopathy among Euthyroid Type 2 Diabetes Patients: A Hospital-Based, Retrospective Study

**DOI:** 10.1155/2022/2552186

**Published:** 2022-01-06

**Authors:** Xiaotong Gao, Xichang Wang, Yifan Zhong, Lei Liu, Weiping Teng, Zhongyan Shan

**Affiliations:** ^1^Department of Endocrinology and Metabolism and the Institute of Endocrinology, The NHC Key Laboratory of Diagnosis and Treatment of Thyroid Diseases, The First Hospital of China Medical University, Shenyang 110001, China; ^2^Department of Ophthalmology, The First Hospital of China Medical University, Shenyang 110001, China

## Abstract

**Background:**

Previous studies have revealed that the variation of thyroid indicators may be associated with the risk of diabetic retinopathy (DR) among euthyroid type 2 diabetes (T2D) patients. But the specific conclusions are currently inconsistent.

**Methods:**

This is a hospital-based retrospective survey. We recruited 1,145 euthyroid T2D patients and checked the thyroid function and fundus photographs. The modified Airlie House classification system was used to categorize the stages of DR. The association between thyroid indicators and different stages of DR was analyzed.

**Results:**

We divided free triiodothyronine (FT3) into tertiles and found that the prevalence of mild nonproliferative DR (NPDR) was significantly higher in T2, compared with T1 (32.0% vs. 25.2%, *p* < 0.05). When FT3 was within the level of T2, FT3 could be an independent risk factor for mild NPDR (OR 1.426, 95% CI (1.031, 1.971), *p* < 0.05). In addition, the prevalence of severe NPDR and proliferative DR (PDR) was significantly higher in thyroglobulin antibody (TgAb) positive group (8.8% vs. 4.1%, *p* < 0.05) and vice versa (33.3% vs. 18.4%, *p* < 0.05). TgAb positivity was also an independent risk factor for severe NPDR and PDR (OR 2.212, 95% CI (1.244, 3.934), *p* < 0.05).

**Conclusions:**

We hardly observed a significant change in DR risk with the elevation or reduction of serum TSH or thyroid hormone within the reference interval. Although the slightly elevated FT3 may be associated to mild NPDR, the extensibility of this result remains to be seen. For T2D patients with euthyroid function, there may be a significant correlation between serum TgAb positivity and severe NPDR and PDR.

## 1. Introduction

Thyroid dysfunction and diabetes are the two most common endocrine diseases in clinical practice. A recent national cross-sectional study has revealed that in mainland China the weighted prevalence of thyroid dysfunction and diabetes have reached up to 15.33% and 12.88%, respectively [1, 2], and a huge amount of evidence have demonstrated the potential association between them [3]. Type 2 diabetes (T2D) is currently the most common type of diabetes, accounting up to 95% of overall diabetes [4]. Several previous studies have demonstrated a significantly higher prevalence of thyroid dysfunction among T2D patients and vice versa [5–11]. Subclinical hypothyroidism (SCH) is recognized as the most common type of thyroid dysfunction [1], and there are currently the most studies on SCH in the relevant investigations on thyroid dysfunction and diabetic complications. Han et al. have meta-analyzed ten studies and found that there was a 2.32-fold increasing risk for SCH, among patients with T2D [12]. Based on the same meta-analysis, the prevalence risks of diabetic nephropathy (DN), diabetic retinopathy (DR), peripheral arterial disease, and neuropathy were also positively associated with SCH [12]. In addition, several studies have found that although the prevalence of hyperthyroidism in T2D patients is far lower than hypothyroidism, it is generally higher than in the normoglycemic population [9, 13, 14].

As for the prevalence risk of T2D and its complications among euthyroid subjects, there are currently few but inconsistent conclusions. Since the variation of thyroid function within the normal range may also be associated with poor glycemic control in T2D patients [15, 16], there must be a potential association between thyroid indicators and T2D complications in euthyroid subjects. However, the evidence is currently scarce, and there are a few discrepancies in the relevant conclusions [17–19]. Therefore, we need to carry out more studies to analyze whether various thyroid indicators are associated with T2D complications among euthyroid subjects.

As the leading cause of moderate to severe blindness in working aged adults, as well as the most common and specific microvascular complication, DR has received more and more extensive attention in recent years [20, 21]. Optimal control of hyperglycemia, hypertension, and dyslipidemia remains the foundation for reduction of DR development and progression [20]. However, in light of the higher prevalence of thyroid dysfunction among T2D patients, it is necessary to explore the potential impact of thyroid indicators on the prevalence and development of DR. There is currently insufficient evidence in this field, especially for euthyroid subjects. In this hospital-based retrospective survey, we conducted an association analysis for euthyroid T2D patients, in order to supplement and innovate the relevant previous conclusions.

## 2. Materials and Methods

### 2.1. Study Participants

The present study was approved by the Medical Ethics Committee of the First Affiliated Hospital of China Medical University. The protocol of this hospital-based retrospective study has been previously described in another article [22]. Briefly speaking, we recruited 2,880 diabetic patients aged ≥ 18 years between February 2012 and November 2018. The diagnostic criteria for diabetes were derived from the 2019 American Diabetes Association (ADA) guidelines [4]. If the patient met one of the four following items, diabetes could be diagnosed: fasting plasma glucose (FPG) ≥ 7.0 mmol/L, glycosylated hemoglobin (HbA_1c_) ≥ 6.5%, 2-hour plasma glucose (2h − PG) ≥ 11.1 mmol/L, with classic symptoms plus random plasma glucose ≥ 11.1 mmol/L. In addition, patients who reported a personal history of diabetes were also recognized as diabetes patients. All patients signed an informed consent form and then completed the questionnaire survey. Information about demographics, personal history of diseases, and medications was all covered by the questionnaire.

**Figure 1:**
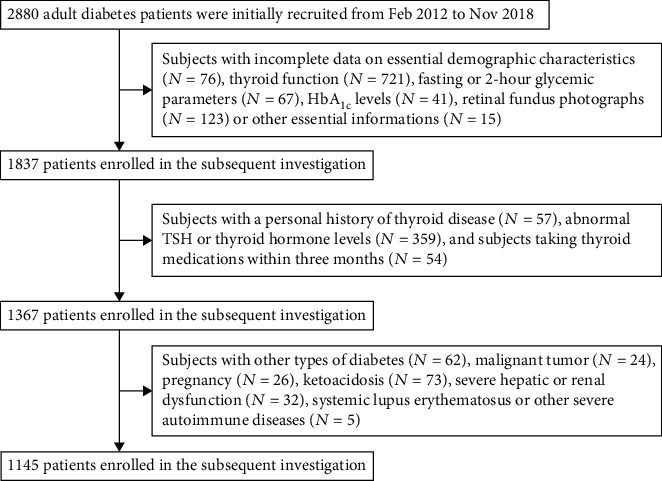
The screening process of the participants.

The screening process of the study population is shown in Figure 1. We first excluded patients with incomplete essential information, and then patients with a personal history of thyroid disease, abnormal TSH or thyroid hormone levels (FT3 or FT4), and those who had taken thyroid drugs within 3 months were also excluded. To avoid the impact of nonthyroid disease syndrome (NTIS) or pregnancy on thyroid function, patients with malignancy, ketoacidosis, severe hepatic or renal failure, other forms of diabetes, or severe autoimmune diseases, as well as pregnant women were also excluded. Finally, 1,145 euthyroid T2D patients were involved in the final analysis.

### 2.2. Clinical and Laboratory Measurement

As previously described [22], weight and height were measured by trained nurses, and body mass index (BMI, kg/m^2^) was calculated as weight divided by the square of the height. Systolic BP (SBP), diastolic BP (DBP), and heart rate (HR) were measured three times with standard mercury sphygmomanometers, and the average was regarded as the final value. After an overnight fasting (≥8 hours), venous blood samples were taken and immediately transferred to the Endocrinology Laboratory of China Medical University for further measurements.

The following fasting biochemical parameters could be queried in the Hospital Information System (HIS), which mainly include high-density lipoprotein (HDL, mmol/L), low-density lipoprotein (LDL, mmol/L), triglyceride (TG, mmol/L), total cholesterol (TC, mmol/L), FPG (mmol/L), fasting insulin (FINS, mU/L), fasting C peptide (FCP, pmol/L), and HbA_1c_ (%). In addition, all the participants went through a 2-hour 75 g oral glucose tolerance test (2h-OGTT). We also recorded the results of 2h-PG, 2h-INS, and 2h-CP after taking the glucose. The specific testing instruments of the above indicators were the same as the previous study [22]. What is more, serum TSH, FT3, FT4, thyroid peroxidase antibody (TPOAb), and TgAb were tested with supersensitive chemiluminescence immunoassay (ARCHITECT system i2000SR, Abbott, Chicago, US). The five thyroid parameters of each patient were also queried by trained nurses from the HIS.

### 2.3. Diagnostic Criteria of Thyroid Dysfunction and Dyslipidemia

As defined by the manufacturer, the reference interval of serum TSH, FT3, FT4, TPOAb, and TgAb was 0.35-4.94 mU/L, 2.43-6.01 pmol/L, 9.01-19.05 pmol/L, ≤5.61 IU/mL, and ≤4.11 IU/mL, respectively. Euthyroid was defined as normal TSH plus normal free thyroid hormone (FT3 and FT4) levels, with or without positive TPOAb or TgAb. The diagnostic criteria for dyslipidemia and hyperuricemia were also defined by the kit. If the subject met all the following four items and was not taking lipid-lowering drugs, it could be considered as normal lipidemia; otherwise, dyslipidemia was diagnosed: HDL ≥ 0.91 mmol/L, LDL ≤ 3.64 mmol/L, TG ≤ 1.7 mmol/L, and TC ≤ 5.72 mmol/L. The upper limit of the reference value of uric acid is 428 *μ*mol/L. If the level of serum uric acid was higher than the above value or the participant was currently taking hypouricemic drugs, then hyperuricemia would be diagnosed.

### 2.4. Assessment and Stratification of DR

A nonmydriatic fundus camera (CR6-45NM; Canon, Tokyo, Japan) was used to evaluate the presence and severity of DR. Two-field fundus examination focusing on the fovea and optic disk was applied by trained ophthalmologists to evaluate the location and extent of lesions. The specific protocol of fundus examination has been previously introduced [22]. According to the Early Treatment for Diabetic Retinopathy Study (ETDRS), the modified Airlie House classification system was referred to evaluate the severity of DR [23], which grouped DR into nonproliferative DR (NPDR) and proliferative DR (PDR). NPDR was further divided into mild, moderate, and severe NPDR.

### 2.5. Statistical Analysis

The above data were input into the Statistical Package for the Social Sciences version 25 (SPSS Inc., Chicago, IL, USA). All *p* values obtained were based on two-tailed tests, with significance level set at 0.05. In the descriptive analysis, continuous variables were described as means and standard deviations (SD), and dichotomous variables were described as numbers and corresponding percentages. Single-sample *t*-test and chi-square test were, respectively, used to compare the differences in continuous and dichotomous variables, both between the two groups with or without DR, as well as between the groups with different levels of thyroid function. Multivariate logistic regression was used to analyze the influence of different thyroid parameters on the prevalence DR.

In order to explore whether different concentrations of serum thyroid indicators are related to the risk of different stages of DR, we divided the thyroid indicators, i.e., TSH, FT3, and FT4, into three tertiles (T1-T3) according to the principle of equalizing the number of people in each group. T2 and T3 were assessed to explore the variation of DR risk with the elevation of the three indicators, compared with T1. In addition, subjects were also divided into two groups according to the values of TPOAb and TgAb (i.e., positive group and negative group).

## 3. Results

### 3.1. General Characteristics of the Patients

As shown in Table 1, the patients were grouped into two groups by the present of DR. The present study included 719 T2D patients without DR and 426 DR patients. Anthropometric, demographic, and biochemical information were listed and compared between patients with and without DR.

We found that T2D patients with DR were significantly older than those without DR (*p* < 0.01). Compared with T2D patients without DR, DR patients had a significantly lower BMI (*p* = 0.04). What is more, the HR and SBP values were both significantly higher in the DR group (*p* < 0.01). However, the difference in the prevalence of hypertension between DR and non-DR patients was not significant.

As for the comparison of serum biochemical indicators (Table 1), we found that the level of plasma HbA_1c_ was significantly higher in the DR group (*p* = 0.04). However, the remaining plasma glycemic indicators did not show significancy between the two groups, whether it was a fasting or a 2-hour indicator. Moreover, regarding the difference in the homeostasis model assessment of insulin resistance (HOMA-IR), DR patients had a slightly but insignificantly higher degree of insulin resistance.

### 3.2. The Changing Patterns of Thyroid Indicators with Different Stages of DR

Due to the extremely low prevalence of PDR (17/1,145), to ensure the reliability of the results of the investigation, patients with severe NPDR or PDR in this study were combined into one group. According to the presentation and severity of retinopathy, the subjects were divided into four groups, and the serum thyroid index levels between the groups were compared.

As shown in Table 2, except for the positive rate of serum TgAb, there was no statistical difference in the remaining four thyroid indicators among subjects in different groups. We found that the positive rate of serum TgAb had a significantly increasing trend (*p* for trend <0.05), with the continuous aggravation of ocular lesions. Moreover, compared with patients without DR, patients with severe NPDR or PDR had a significantly higher positive rate of TgAb (33.3% vs. 18.4%, *p* < 0.05).

### 3.3. The Changing Patterns of DR Prevalence with Thyroid Indicators

Serum TSH, FT4, and FT3 were all divided into tertiles, and the DR prevalence corresponding to different tertiles was calculated; at the same time, DR prevalence was also calculated according to the presentation of positive serum thyroid antibodies. The subgroups with the lowest levels of hormones or negative antibodies were regarded as the reference.

**Figure 2:**
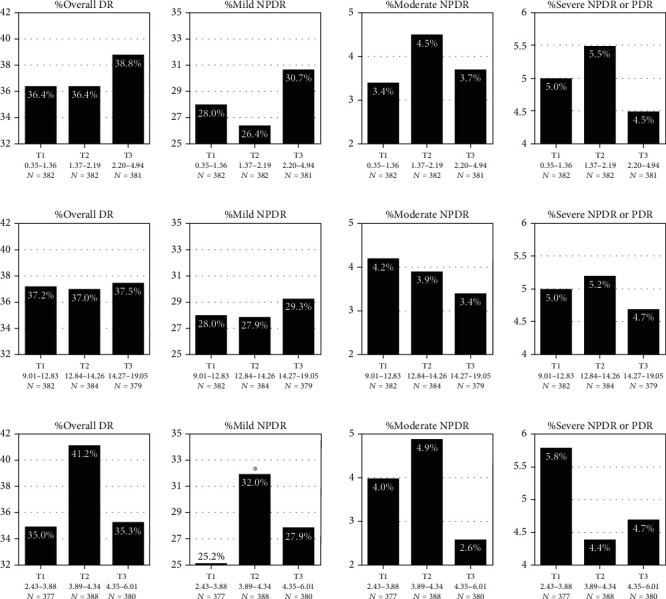
The variation of different stages of DR prevalence with serum TSH (a), FT4 (b), and FT3 (c). DR: diabetic retinopathy; NPDR: nonproliferative diabetic retinopathy; PDR: proliferative diabetic retinopathy; TSH: thyroid-stimulating hormone; FT4: free thyroxine; FT3: free triiodothyronine. The stratification of the thyroid indicators is stated in the figure, and the unit of TSH, FT4, and FT3 should be mU/L, pmol/L, and pmol/L, respectively. The ^∗^ indicates that the corresponding prevalence was significantly higher than the control group (T1) (*p* < 0.05).

As shown in the (a) and (b) in Figure 2, we found that the prevalence of different stages of DR or overall DR was not significantly higher or lower, with the increase of TSH or FT4. The prevalence of DR corresponding to different levels of TSH or FT4 was relatively comparable. In addition, the overall DR prevalence corresponding to different levels of FT3 was also similar. However, the prevalence of mild NPDR in the second tertile (T2) of FT3 was significantly higher (32.0% vs. 25.2%, *p* < 0.05), suggesting that the prevalence of mild NPDR was significantly higher in T2D patients with slightly elevated FT3 within reference interval (3.89-4.34 pmol/L).

**Figure 3:**
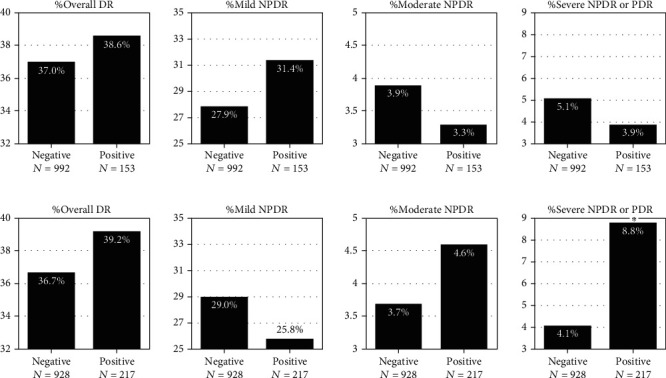
The variation of different stages of DR prevalence with serum TPOAb (a) and TgAb (b). DR: diabetic retinopathy; NPDR: nonproliferative diabetic retinopathy; PDR: proliferative diabetic retinopathy; TPOAb: thyroid peroxidase antibody; TgAb: thyroglobulin antibody. The ^∗^ indicates that the corresponding prevalence in the positive group was significantly higher than the negative group (*p* < 0.05).

Regarding the prevalence of DR under different thyroid autoantibody titers, we found that the DR prevalence did not significantly increase or decrease for T2D patients with positive TPOAb (Figure 3(a)). On the other side, the prevalence of severe NPDR or PDR was significantly higher in TgAb-positive patients (8.8% vs. 4.1%, *p* < 0.05) (Figure 3(b)), while the prevalence of other stages of DR did not show significancy.

### 3.4. Regression Analysis between Thyroid Function and DR Risks

In order to further confirm the association between the thyroid indicators and different grades of DR risk, univariable and multivariable regression analyses were applied to explore the abovementioned differences in DR prevalence (Table 3). The grouping of thyroid indicators was similar to the above description, and then, the groups with the lowest tertile and the antibody-negative groups were set as the reference.

As shown in Table 3, the T2 of FT3 correlated with the risk of mild NPDR (OR 1.426 (1.031, 1.971), *p* < 0.05). What is more, TgAb positivity was also significantly associated with the risk of severe NPDR or PDR (OR 2.212 (1.244, 3.934), *p* < 0.05). The above correlations were both significant in both model 1 and model 2. However, our results were unable to deduce the significant relationships between the variations in TSH or FT4 within the reference range or the TPOAb positivity and the risk of DR. In addition, we also explored whether the risk of each stage of DR and overall DR would change significantly with the linear changes of TSH, FT4, and FT3. However, with per SD-increase in the abovementioned three indicators, there was no significant variation in the risk of DR.

## 4. Discussion

In this hospital-based retrospective survey, 1,145 T2D patients with normal thyroid function were included. We found that a slight increase in FT3 (3.89-4.34 pmol/L) within the reference range may be significantly associated with the risk of mild NPDR. Patients with a slightly higher FT3 within the specific range had a significantly higher prevalence of mild NPDR. It should be emphasized that although the mild increase in serum FT3 is significantly correlated with mild NPDR, this conclusion does not have much significance in clinical practice. More research is needed to explore the potential association between FT3 levels beyond the reference interval and different stages of DR. In addition, we found that the prevalence of severe NPDR or PDR in TgAb-positive patients was also significantly higher and vice versa. TgAb positivity may also be an independent risk factor for severe DR in euthyroid T2D patients.

The anthropometric and biochemical information were compared in patients with or without DR. We found that compared with non-DR patients, T2D patients with DR are significantly older and have significantly lower levels of BMI, and there are also slight differences in the prevalence of hypertension between the two groups, especially in the significantly higher SBP in DR patients. Compared with patients without DR, patients with DR had a slightly increased prevalence of dyslipidemia and hyperuricemia. In addition, similar to our inherent knowledge that T2D patients with complications generally have worse control of blood glucose than noncomplicated patients, the DR group showed a higher HbA_1c_, and fasting and 2-hour plasma indicators, as well as insulin resistance level also showed insignificant but slight worsen trend.

As mentioned above, several previous studies have been conducted on DR risk and thyroid function, especially SCH, but the conclusion is not clear yet. Similar to the conclusions of several individual studies [24–27], Han et al. have meta-analyzed ten Chinese studies and found that T2D patients with SCH were more likely to be accompanied with DR, and the risk could be increased by 42% [12]. However, a recent Indian study found that not only DR but other T2D complications such as nephropathy, neuropathy, or cardiovascular disease were not significantly related with any forms of thyroid dysfunction [28]. What is more, several studies also concluded that supranormal or high-normal TSH level was not related with DR, while the risk of nephropathy showed significance [29–31].

Therefore, currently, we have insufficient evidence to support an explicit relationship between thyroid dysfunction (especially SCH) and DR. Compared with the above studies, there are fewer studies on thyroid function and DR risk among euthyroid T2D subjects. In a similar hospital-based retrospective study [32], Zou et al. revealed that the prevalence of DR decreased significantly with the increase of serum FT3 level among euthyroid subjects, and the level of FT3 also decreased significantly with the severity of DR. However, the level of TSH or FT4 or the positivity of TPOAb showed no significant trends. Different from the above findings, Kong et al. found that the DR prevalence showed a significantly decreasing trend across the tertiles of FT4, plus a significantly increasing trend across the tertiles of TSH. However, the level of FT3 hardly showed any significance regarding DR or PDR [33]. We speculate that the scales of the previous studies were small, so sampling bias might lead to the inconsistency of the conclusions. In addition, the kits used in various studies are different, so the inconsistent reference intervals for TSH, FT3, or FT4 might also contribute to inconsistent conclusions. Finally, the relevant studies so far are based on hospital-based retrospective populations, so untreated or undiagnosed T2D patients were ignored. It remains to be further explored that whether these evidence truly revealed the association between thyroid indicators and DR risk among euthyroid T2D patients.

Compared with the previous findings, the results of the present research are somewhat different. The prevalence of overall or each stage of DR showed no significant difference across the tertiles of TSH or FT4. However, the prevalence of mild NPDR in the T2 of FT3 was significantly higher than in the T1 of FT3. The following regression analysis further confirmed the above difference in prevalence. We found that when serum FT3 was in the range of T2 (3.89-4.34 mmol/L), the risk of mild NPDR was significantly associated with it. However, when FT3 continues to rise to the level of T3, the above association has no statistical significance. Unlike the previous negative correlation, we consider that the slight increase in FT3 within the reference range may be positively correlated with the risk of mild NPDR. Although previous meta-analysis found that SCH might be related to the risk of DR, there are currently very few relevant studies carried out in euthyroid T2D patients. Based on the evidence from previous studies, we cannot conclude whether changes in thyroid hormones within the reference range will affect the risk of DR in euthyroid patients with T2D. Our study proposed for the first time a significant association between mildly elevated FT3 and mild DR in euthyroid T2D patients, which is a good supplement to the existing evidence.

Because of the common cooccurrence of autoimmune diseases [34], there have been a number of relevant studies on thyroid autoimmune and DR in T1D patients [35], but we could hardly find any explicit findings on T2D patients. In this study, we found a significant positive correlation between TgAb positivity and the risk of severe NPDR or PDR among euthyroid T2D patients. This is the first ever explicit conclusion about the relationship between thyroid autoimmunity and T2D-DR. Therefore, more future evidence is required to confirm such a correlation between thyroid autoimmune and the complications of T2D. A variety of inflammatory cytokines in serum have been confirmed to be closely related to the occurrence and development of T2D-DR [36–38]. At the same time, a large number of studies also showed that a variety of circulating cytokines involving in the occurrence and development of thyroid autoimmunity [39–41]. We speculate that the above two groups of pathological processes might overlap or promote each other, which induces a higher TgAb titer in patients with severe DR and vice versa. In view of the fact that there are few basic investigations in the related fields at present, more research is needed for in-depth exploration in the future. Although the manifestations of autoimmune thyroid disease (AITD) are generally ignored in practice, it has a certain impact on long-term thyroid function. Given that severe DR patients have a significantly higher TgAb positive rate, it is necessary to discuss in more depth whether it is essential to screen for serum thyroid autoantibodies in T2D patients with severe DR.

This study has some limitations. Firstly, this is a retrospective survey, so we cannot know the causal relationship between thyroid function and DR. Secondly, the current treatment rate of diabetes in mainland China is less than half [2]. Therefore, whether this hospital-based survey reflects the actual association between thyroid function and DR risk in general T2D patients remains unknown.

## 5. Conclusions

In summary, we hardly found that the risks of various stages of DR showed a significant upward or downward trend with serum TSH or thyroid hormone within the reference interval. Although mildly elevated FT3 may be associated with an increased risk of mild NPDR, the extensibility of this finding remains to be explored. In addition, there is a significant positive correlation between TgAb positivity and severe DR. The positive rate of TgAb in severe NPDR and PDR patients is much higher than that in non-DR T2D patients and vice versa. Further prospective studies are needed to get enough evidence on the association between the thyroid autoantibodies and the presence or development of severe DR.

## Figures and Tables

**Table 1:** General characteristics of participants stratified by the presence of DR.

Number (*N*, %)	Total	Non-DR	DR	*p* value
	1,145	719	426	
Sex (female, %)	494 (43.1%)	305 (42.4%)	189 (44.4%)	0.52
Age (years)	56.30 ± 13.32	55.45 ± 14.11	57.73 ± 11.74	<0.01
T2D duration (>10 years, %)	726 (63.4%)	443 (61.6%)	283 (66.4%)	0.10
Smoking (*N*, %)	1,017 (88.8%)	635 (88.3%)	382 (89.7%)	0.48
Drinking (*N*, %)	1004 (87.7%)	628 (87.3%)	376 (88.3%)	0.64
BMI (kg/m^2^)	25.62 ± 3.78	25.75 ± 3.94	25.40 ± 3.50	0.04
HR (per min)	81.34 ± 11.19	80.78 ± 10.82	82.30 ± 11.74	<0.01
SBP (mmHg)	135.15 ± 20.28	133.77 ± 19.25	137.49 ± 21.74	<0.01
DBP (mmHg)	82.50 ± 11.88	82.08 ± 11.75	83.19 ± 12.09	0.06
Hypertension (*N*, %)	579 (50.6%)	348 (48.4%)	231 (54.2%)	0.06
Hyperuricemia (*N*, %)	113 (9.9%)	39 (9.2%)	74 (10.3%)	0.53
Dyslipidemia (*N*, %)	604 (52.8%)	373 (51.9%)	231 (54.2%)	0.44
HbA_1c_ (%)	8.64 ± 2.43	8.54 ± 2.37	8.80 ± 2.52	0.04
FPG (mmol/L)	9.25 ± 3.89	9.24 ± 3.47	9.26 ± 4.12	0.92
2h-PG (mmol/L)	17.47 ± 5.89	17.24 ± 5.18	17.61 ± 6.27	0.14
FINS (mU/L)	14.55 ± 49.88	14.25 ± 51.14	15.06 ± 47.73	0.73
2h-INS (mU/L)	32.57 ± 28.04	31.77 ± 25.10	33.91 ± 32.41	0.18
FCP (pmol/L)	651.81 ± 531.62	649.36 ± 514.26	655.96 ± 560.28	0.81
2h-CP (pmol/L)	1727.33 ± 1201.89	1732.11 ± 1223.63	1792.37 ± 1165.27	0.19
HOMA-IR	6.05 ± 20.38	5.67 ± 15.48	6.70 ± 26.70	0.43

T2D: type 2 diabetes; DR: diabetic retinopathy; BMI: body mass index; HR: heart rate; SBP: systolic blood pressure; DBP: diastolic blood pressure; HbA_1c_: glycosylated hemoglobin; FPG: fasting plasma glucose; FINS: fasting insulin; FCP: fasting C peptide; HOMA-IR: homeostasis model assessment of insulin resistance. The diagnosing criteria of hypertension were SBP ≥ 140 mmHg or DBP ≥ 90 mmHg or current administration of antihypertensive drugs. Smoking and drinking were defined as a personal history of smoking/drinking or current smoking/drinking, regardless of the frequency. Dyslipidemia and hyperuricemia were defined by the reference interval provided by the kit, and subjects currently taking lipid-lowering or hypouricemic drugs were also regarded as dyslipidemia or hyperuricemia patients. HOMA-IR was calculated as follows: HOMA − IR = FPG × FINS/22.5.

**Table 2:** Thyroid indicators corresponding to different severities of DR.

	Non-DR	Mild NPDR	Moderate NPDR	Severe NPDR or PDR
Number	719	325	44	57
TSH (mU/L)	1.92 ± 0.99	1.99 ± 1.08	1.99 ± 1.14	1.83 ± 0.88
FT4 (pmol/L)	13.58 ± 1.72	13.72 ± 1.78	13.57 ± 1.70	13.57 ± 1.79
FT3 (pmol/L)	4.10 ± 0.61	4.11 ± 0.62	4.06 ± 0.59	4.10 ± 0.74
pTPOAb (*N*, %)	94 (13.1%)	48 (14.8%)	5 (11.4%)	6 (10.5%)
pTgAb (*N*, %)^b^	132 (18.4%)	56 (17.2%)	10 (22.7%)	19 (33.3%)^a^

TSH: thyroid-stimulating hormone; FT4: free thyroxine; FT3: free triiodothyronine; pTPOAb: positive thyroid peroxidase antibody; pTgAb: positive thyroglobulin antibody; DR: diabetic retinopathy; NPDR: nonproliferative diabetic retinopathy; PDR: proliferative diabetic retinopathy. Data are expressed as mean ± SD or number and proportion. The superscript a indicates that the value in the corresponding group is significantly higher than the non-DR group (*p* < 0.05). The superscript b indicates that the variation of the thyroid indicator shows a significantly upward trend.

**Table 3:** Associations between thyroid indicators and various stages of DR risk.

		Overall DR	Mild NPDR	Moderate NPDR	Severe NPDR or PDR
TSH	T1 (ref)	1	1	1	1
	T2 (model 1)	1.000 (0.745, 1.343)	0.924 (0.672, 1.271)	1.322 (0.633, 2.761)	1.111 (0.588, 2.102)
	T2 (model 2)	0.990 (0.734, 1.333)	0.917 (0.665, 1.264)	1.274 (0.607, 2.674)	1.133 (0.596, 2.152)
	T3 (model 1)	1.110 (0.828, 1.489)	1.139 (0.834, 1.556)	1.083 (0.502, 2.335)	0.892 (0.456, 1.744)
	T3 (model 2)	1.062 (0.789, 1.431)	1.116 (0.814, 1.529)	0.954 (0.437, 2.081)	0.851 (0.431, 1.680)
	Per SD increase (model 1)	1.055 (0.938, 1.187)	1.081 (0.954, 1.225)	1.049 (0.783, 1.404)	0.893 (0.679, 1.174)
	Per SD increase (model 2)	1.043 (0.926, 1.175)	1.072 (0.945, 1.216)	1.017 (0.757, 1.367)	0.891 (0.675, 1.176)

FT4	T1 (ref)	1	1	1	1
	T2 (model 1)	0.992 (0.740, 1.330)	0.993 (0.724, 1.361)	0.930 (0.453, 1.909)	1.050 (0.551, 2.000)
	T2 (model 2)	1.035 (0.768, 1.395)	1.030 (0.748, 1.418)	0.973 (0.471, 2.010)	1.073 (0.562, 2.050)
	T3 (model 1)	1.013 (0.755, 1.358)	1.064 (0.777, 1.458)	0.813 (0.385, 1.713)	0.953 (0.492, 1.845)
	T3 (model 2)	0.983 (0.728, 1.328)	1.069 (0.776, 1.471)	0.808 (0.379, 1.726)	0.826 (0.421, 1.620)
	Per SD increase (model 1)	1.035 (0.967, 1.109)	1.047 (0.973, 1.127)	0.985 (0.827, 1.172)	0.983 (0.843, 1.147)
	Per SD increase (model 2)	1.028 (0.959, 1.102)	1.042 (0.968, 1.122)	0.984 (0.826, 1.173)	0.968 (0.831, 1.128)
FT3	T1 (ref)	1	1	1	1
	T2 (model 1)	1.302 (0.972, 1.745)	1.394 (1.017, 1.912)^c^	1.243 (0.622, 2.483)	0.739 (0.386, 1.416)
	T2 (model 2)	1.349 (0.997, 1.824)	1.426 (1.031, 1.971)^c^	1.146 (0.566, 2.320)	0.840 (0.431, 1.637)
	T3 (model 1)	1.011 (0.750, 1.363)	1.148 (0.831, 1.586)	0.652 (0.289, 1.471)	0.802 (0.423, 1.521)
	T3 (model 2)	1.031 (0.757, 1.405)	1.179 (0.844, 1.647)	0.588 (0.254, 1.363)	0.846 (0.434, 1.646)
	Per SD increase (model 1)	1.016 (0.837, 1.235)	1.051 (0.853, 1.295)	0.876 (0.538, 1.425)	0.973 (0.632, 1.499)
	Per SD increase (model 2)	1.016 (0.829, 1.246)	1.061 (0.853, 1.318)	0.744 (0.445, 1.244)	1.047 (0.673, 1.628)

TPOAb	Negative	1	1	1	1
	Positive (model 1)	1.069 (0.753, 1.517)	1.180 (0.816, 1.706)	0.826 (0.320, 2.128)	0.753 (0.318, 1.786)
	Positive (model 2)	1.085 (0.761, 1.547)	1.191 (0.822, 1.726)	0.859 (0.332, 2.226)	0.749 (0.314, 1.785)

TgAb	Negative	1	1	1	1
	Positive (model 1)	1.108 (0.818, 1.502)	0.852 (0.609, 1.192)	1.270 (0.618, 2.613)	2.247 (1.269, 3.981)^c^
	Positive (model 2)	1.112 (0.818, 1.511)	0.849 (0.606, 1.190)	1.343 (0.649, 2.780)	2.212 (1.244, 3.934)^c^

Model 1: crude; Model 2: adjusted for age, sex, BMI, SBP, and HbA_1c_. The superscript c indicates that the *p* value of corresponding OR is less than 0.05.

## Data Availability

The database of the study is available on request to Prof. Lei Liu (email address: liuleijiao@163.com).
